# Taxa of the Nasal Microbiome Are Associated with Influenza-Specific IgA Response to Live Attenuated Influenza Vaccine

**DOI:** 10.1371/journal.pone.0162803

**Published:** 2016-09-19

**Authors:** Hannah M. Salk, Whitney L. Simon, Nathaniel D. Lambert, Richard B. Kennedy, Diane E. Grill, Brian F. Kabat, Gregory A. Poland

**Affiliations:** 1 Mayo Vaccine Research Group, Mayo Clinic, Rochester, MN, 55905, United States of America; 2 Division of Biostatistics, Mayo Clinic, Rochester, MN, 55905, United States of America; University of South Dakota, UNITED STATES

## Abstract

Live attenuated influenza vaccine (LAIV) has demonstrated varying levels of efficacy against seasonal influenza; however, LAIV may be used as a tool to measure interactions between the human microbiome and a live, replicating virus. To increase our knowledge of this interaction, we measured changes to the nasal microbiome in subjects who received LAIV to determine if associations between influenza-specific IgA production and the nasal microbiome exist after immunization with a live virus vaccine. The anterior nares of 47 healthy subjects were swabbed pre- (Day 0) and post- (Days 7 and 28) LAIV administration, and nasal washes were conducted on Days 0 and 28. We performed next-generation sequencing on amplified 16s rRNA genes and measured mucosal influenza-specific IgA titers via enzyme-linked immunosorbent assay (ELISA). A significant increase in alpha diversity was identified (Observed, CHAO, and ACE) between Days 7 vs 0 (p-values = 0.017, 0.005, 0.005, respectively) and between Days 28 vs 0 (p-values = 0.054, 0.030, 0.050, respectively). Several significant associations between the presence of different microbial species, including *Lactobacillus helveticus*, *Prevotella melaninogenica*, *Streptococcus infantis*, *Veillonella dispar*, *and Bacteroides ovatus*, and influenza-specific H1 and H3 IgA antibody response were demonstrated. These data suggest that LAIV alters the nasal microbiome, allowing several less-abundant OTUs to establish a community niche. Additionally, specific alterations in the nasal microbiome are significantly associated with variations in influenza-specific IgA antibody production and could be clinically relevant.

## 1. Introduction

Influenza causes significant economic and health-related burdens worldwide, resulting in an estimated three to five million cases of serious illness and 250,000–500,000 deaths annually [1]. Annual influenza vaccination is the best available method of protection against the disease; however, the protection offered by the vaccine is imperfect.

It is known that commensal bacteria are important for maintaining immune homeostasis in the gut [2,3], but a growing amount of evidence demonstrates that these bacteria may also play immunomodulatory roles in non-intestinal mucosal surfaces, such as the respiratory mucosa [4,5], and even systemically [5,6,7]. In fact, even small amounts of inoculum introduced to the nasal cavity are later detectable in the gastrointestinal tract[8]; therefore, because we know that the mucosal immune system acts as a sensor of foreign antigens, it has been suggested that alterations in the microbiota of these mucosal surfaces could impact the immune responses to foreign antigens [7].

Influenza-specific studies have shown that commensal bacteria influence the immune responses to both influenza infection [4] and vaccination [9,10,11]. In particular, it has been suggested that commensal bacteria may enhance these immune responses through inflammasome activation [4]; providing ligands for pattern recognition receptors such as TLRs [9]; increasing NK cell activity [12]; or inhibiting the conversion of Tregs in the gut mucosa, resulting in prolonged suppression of adaptive immunity [13]. Further evidence of this relationship between the microbiota and the immune response to influenza infection and vaccination includes the positive effect of prebiotics and probiotics on immune responses to these perturbations [12,14].

To date, studies have not addressed the relationship between the human nasal microbiota and localized, influenza vaccine-specific IgA mucosal response to intranasally administered live attenuated influenza vaccine (LAIV). Here, we examine the nasal microbiome of 47 subjects using next-generation sequencing before (Day 0) and after (Days 7 and 28) receipt of the 2012–2013 seasonal LAIV. We also investigate correlations between subjects’ microbiome composition and the localized, mucosal immune responses to the influenza virus.

## 2. Materials and Methods

### 2.1 Study subjects and sample collection

The study cohort was comprised of 47 healthy, young adult subjects ranging in age from 18–30 years. An anterior nares swab was collected from each study participant prior to vaccination (Day 0) with a Catch-All Collection Swab (Epicenter; Madison, WI). The swab was saturated in TE buffer for 20 seconds, and the buffer was frozen at -20°C until further processing. Each subject was then immunized with a single dose of the FluMist® 2012–2013 seasonal intranasal LAIV (Medimmune, LLC.; Gaithersburg, MD), containing the A/California/07/2009 (H1N1), A/Victoria/361/2011 (H3N2), and B/Wisconsin/1/2010-like influenza virus strains. Nares swabs were again collected on Day 7 and Day 28 post-immunization and processed in the manner described above. After collection of anterior nares swabs, nasal washes were performed with 10 mL of sterile sodium chloride solution on Days 0 and 28. Briefly, 5 mL of sterile 0.9% sodium chloride solution was introduced into the right nostril and held for 10 seconds. Collection of the sample occurred as the subject flexed his/her neck and exhaled through the nose. This process was repeated for the left nostril, and samples were subsequently stored at 4°C until further processing.

All subjects reported good health prior to enrollment in the study. Exclusion criteria included prior immunization with the 2012–2013 influenza vaccine, status as a current smoker, sufferers of eye or nose allergies, or possession of an ornamental piercing in either nostril, or conditions for which LAIV is contraindicated by the Advisory Committee on Immunization Practices of the U.S. Department of Health and Human Services (this included, but was not limited to, asthma; immunosuppression; moderate or severe illnesses with or without fever; and chronic pulmonary, cardiovascular, renal, hepatic, neurological/neuromuscular, hematologic, or metabolic disorders). One subject was excluded from analysis due to the development of sinus infection-like symptoms after vaccination. This study was approved by the Mayo Clinic Institutional Review Board and written informed consent was obtained from each subject.

### 2.2 Influenza-specific IgA quantification

Enzyme-linked immunosorbent assays (ELISAs) were conducted on nasal wash samples (Days 0 and 28) to quantify each subject’s mucosal IgA antibody titers against the influenza-specific hemagglutinin H1 and H3 glycoproteins separately (glycoproteins received from Influenza Reagent Resource; Manassas, VA). Briefly, 96-well Immulon 4 HBX ELISA plates (Thermo Scientific; Pittsburgh, PA) were coated with 100 μl of purified hemagglutinin protein (1 μ/ml) from either influenza A/California/07/2009 (H1N1) or influenza A/Victoria/361/2011 (H3N2) virus and stored overnight at 4°C. Plates were washed with phosphate buffered saline (PBS) and blocked with 200 μl of 1x ELISA Diluent (eBioscience; San Diego, CA) for 1 hour at room temperature. Samples were serially diluted two-fold with 1x ELISA Diluent until a dilution of 1:1,280 was reached. Subsequently, 100 μl of diluted samples were added to the coated ELISA plates. Influenza A virus positive IgG control and an influenza A virus negative control were used (MBL International; Woburn, MA). The plates were incubated at 4°C overnight. After three washes with PBS containing 0.05% tween (PBS-T) the following day, HRP-conjugated anti-human IgA antibody (Abcam; Cambridge, MA) was added to the subject samples and HRP-conjugated IgG antibody (Abcam; Cambridge, MA) was added to the positive and negative controls. The plates were incubated at room temperature for one hour and washed three times with PBS-T before the addition of 1x TMB substrate (eBioscience; San Diego, CA). Samples were allowed to develop for 10 minutes or until the positive control yielded a deep blue hue. Development was terminated by administering 30 μl/well of 2N sulfuric acid. Absorbance was read at 450 nm. Titers were calculated as positive/negative (P/N) ratio ≥ 2. This ratio was used as an exploratory quantitation of the localized mucosal response to vaccination with LAIV, and not considered a correlate of protection. A two-fold increase in influenza-specific IgA titers from baseline has been proposed by others to represent a positive result [15,16].

### 2.3 Isolation of rRNA genes and next-generation sequencing

Isolation of rRNA genes and next-generation sequencing was performed in a manner similar to the methods published by Seto *et al*. [17]. Briefly, DNA was isolated from the specimens obtained by anterior nares swabs (Days 0, 7, and 28) using the PowerSoil® DNA Isolation Kit (MO BIO Laboratories, Inc.; Carlsbad, CA), and isolated DNA was used as a template for a polymerase chain reaction (PCR). The bacterial 16s rRNA genes were amplified using a 357F forward primer for all samples and a unique, bar-coded 926R reverse primer for each individual sample. Amplified PCR products were purified using QIAquick PCR Purification Kit (Qiagen; Valencia, CA) and visualized using an Agilent 2200 TapeStation system (Agilent Technologies; Santa Clara, CA). The correct amplicon (approximately 700 basepairs in length) was visualized under UV light and extracted with a QIAquick Gel Extract Kit (Qiagen; Valencia, CA), and a final concentration was determined using a Qubit 2.0 Fluorometer. 16S rRNA sequencing was performed using the Illumina MiSeq platform and the MiSeq Reagent Kit v2 (2 x 250 reads, 500 cycles; Illumina, Inc.; San Diego, CA). From this data, taxonomy was assigned and phylogenetic trees were constructed using the Illinois Mayo Taxon Organization from RNA Dataset Operations (IM-TORNADO) pipeline [18].

### 2.4 Statistical analysis

Alpha diversity was calculated using four different measures using the “phyloseq” package in R [19]. The observed number of OTUs, Chao’s estimator, [20] and ACE estimator [21,22] provides estimates of species richness, while the Shannon diversity index accounts for both species evenness and richness [23]. Differences over time in each of the alpha diversity measurements were tested with contrast statements sfrom mixed effect linear regression models, which account for within-subject correlation. For analysis purposes, H1 and H3 IgA titer values were analyzed on the log2 scale. Linear regression models were used to assess the effect of alpha diversity on the H1 IgA and H3 IgA Day 28 response variables, adjusting for the baseline H1 IgA and H3 IgA values.

Operational taxonomic units (OTUs)–sequence clusters defined based on DNA sequence similarity at a conserved locus [24]–were used to report sequencing data. Analysis focused on the 49 OTUs that had values in at least 25% of the samples. These OTUs were normalized using relative log expression (RLE) [25,26]. The normalized values were used in separate linear regression models to test for associations with the log2 Day 28 response to H1 and H3; all models were adjusted to the baseline values of H1 and H3. Multivariate analysis was done using penalized regression [27]; this method includes all 49 OTUs as covariates and uses cross-validation to select the model that has the minimum mean square error. Results are reported for OTUs with known species.

Penalized cross-validated regression models, which provide a robust estimate of the combined influence of the taxa on the response to vaccination, were used in lieu of principal component analysis (PCA) because PCA reduces the Taxa (OTUs) to a single point and loses information that may be important for interpretation. The penalized regression models use cross-validation, adjust the coefficients so that the results are robust, and provide a model where the important species associated with the response to vaccination can be readily identified.

## 3. Results

### 3.1 Subject demographics

A total of 47 subjects participated in this study, of whom 27 were female and 20 were male (**Table 1**). The average age at time of enrollment was 21.0 years, with a range of 18–30 years. Subjects self-reported racial/ethnic classifications, which yielded a cohort consisting of 43 Caucasians (91.5%), 3 African Americans (6.4%), and one who self-reported a racial/ethnic classification of “other” (2.1%).

**Table 1:** Subject Demographics.

Variable	No. of Subjects	Measure
Age in years (median, IQR*)	47	21.0 (18.4; 23.6)
Gender (N, %):Female;Male	47	27 (57.4%);20 (42.6%)
Race (N, %):Caucasians;Blacks;Others	47	43 (91.5%)3 (6.4%)1 (2.1%)

*IQR, interquartile range

### 3.2 IgA response to influenza H1 and H3 proteins

Subjects’ mucosal IgA titers against recombinant H1 and H3 influenza proteins were measured by ELISA, as detailed above. A positive response was considered to be at least a two-fold increase in IgA titer from Day 0 to Day 28 post-vaccination. The median baseline IgA titers were 80 (IQR: 60, 160) and 160 (IQR: 80, 320) for H1 and H3 proteins, respectively, indicating that many subjects had preexisting immunity, likely due to prior wild virus exposure and/or previous vaccination. Of the 47 subjects who participated in the study, five subjects had a positive response to the H1 protein only, four subjects had a positive response to the H3 protein only, and 10 subjects responded positively to both proteins (**Table 2**). The proportion of positive H1 responses is comparable to those observed by others for H1 IgA titers. [15] Studies evaluating influenza H3-specific IgA titers could not be found. The remaining 28 subjects did not have a positive IgA response to either hemagglutinin protein within the vaccine.

**Table 2:** Hemagglutinin Response Matrix.

		H3	
		- Response	+ Response
**H1**	- Response	28	4
	+ Response	5	10

### 3.3 Change in microbiome alpha diversity post-LAIV

**Fig 1:**
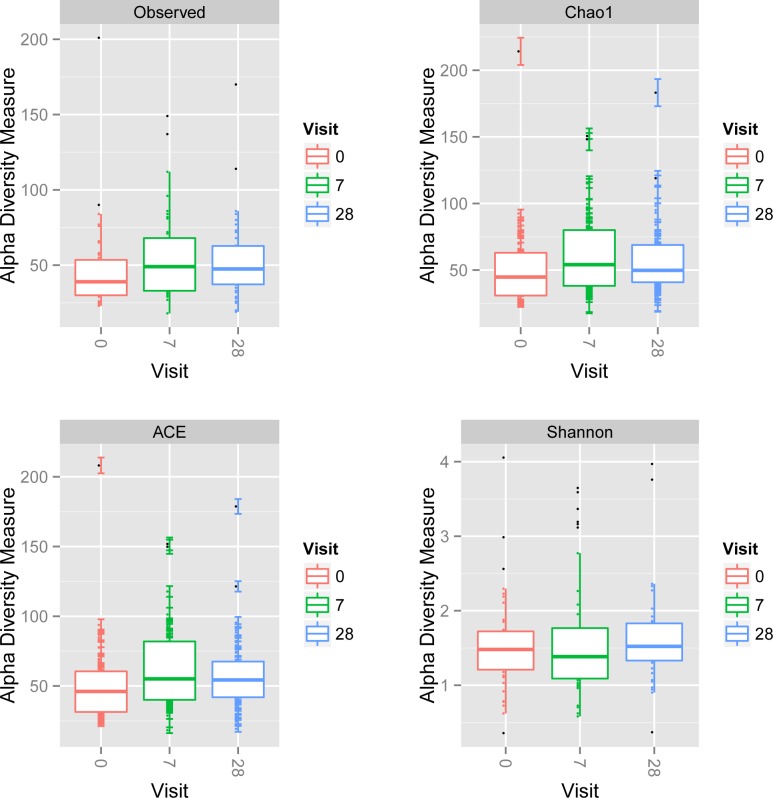
Changes in alpha diversity over time. Observed OTUs, Day 7 vs. Day 0, p = 0.017 and Day 28 vs. Day 0, p = 0.005; Chao estimator, Day 7 vs. Day 0, p = 0.005 and Day 28 vs. Day 0, p = 0.030; ACE estimator, Day 7 vs. Day 0, p = 0.005 and Day 28 vs. Day 0, p = 0.050. There were no significant differences for Shannon’s Diversity Index. Box-and-whiskers plots showing four measures of Alpha Diversity of anterior nares microbial community for each study visit (Day 0, Day 7 and Day 28).

Mixed effect linear regression models were used to assess differences in alpha diversity over time (Fig 1, Table 3) for our four measures of alpha diversity. A significant increase in alpha diversity of the nasal microbiome was identified between Day 7 vs Day 0 by Observed, Chao, and ACE methods (p-values = 0.017, 0.005, and 0.005, respectively). Additionally, a significant increase in alpha diversity was identified between Day 28 vs Day 0 in the Chao analysis (p-value = 0.0297); this increase was on the cusp of significance in both the Observed and ACE analyses (p-values = 0.054 and 0.050, respectively). The Shannon diversity index failed to identify a significant change in alpha diversity between any timepoints.

**Table 3:** Changes in Alpha Diversity Over Time.

Diversity Measure	Comparison	Estimate	Standard Error	T-Statistic	p-Value
Observed	Day 7 vs 0	11.61	4.74	2.45	0.017
	Day 28 vs 0	9.11	4.66	1.96	0.054
	Day 28 vs 7	-2.50	4.62	-0.54	0.590
Chao	Day 7 vs 0	14.82	5.12	2.90	0.005
	Day 28 vs 0	11.14	5.03	2.21	0.023
	Day 28 vs 7	-3.68	4.99	-0.74	0.462
ACE	Day 7 vs 0	14.40	5.03	2.86	0.005
	Day 28 vs 0	9.84	4.95	1.99	0.050
	Day 28 vs 7	-4.56	4.91	-0.93	0.356
Shannon	Day 7 vs 0	0.15	0.12	1.24	0.220
	Day 28 vs 0	0.13	0.12	1.08	0.284
	Day 28 vs 7	-0.02	0.12	-0.18	0.857

Furthermore, an association was identified between H3 IgA response at Day 28, adjusted for H3 IgA antibody response at Day 0, and alpha diversity at Day 0 (Observed p-value = 0.029, Chao p-value = 0.024, ACE p-value = 0.026). Again, this association was not significant for the Shannon metric (data not shown). We did not observe significant beta diversity as characterized by UniFrac distances at baseline and Day 28 with response to vaccination measured by H1 or H3 IgA antibody titers.

### 3.4 Relationship between taxa levels and influenza-specific H1 and H3 IgA titers

Linear regression models for each OTU identified significant positive associations between influenza H1 antibody titer and levels of the following bacterial species at Day 0 and/or post-vaccination timepoints: *Lactobacillus helveticus*, *Prevotella melaninogenica*, *Streptococcus infantis*, *Veillonella dispar*, and *Bacteroides ovatus* (**Table 4**).

**Table 4:** Influenza H1 IgA Titers as Functions of Species at Specified Timepoints.

Species	Timepoint*	Estimate	Standard Error	t value	Pr(>|t|)
*Veillonella dispar*	Day 0	-0.168	0.064	-2.63	0.012
*Streptococcus infantis*	Day 7 –Day 0	0.170	0.060	2.845	0.008
*Prevotella melaninogenica*	Day 7	0.170	0.059	2.888	0.006
*Lactobacillus helveticus*	Day 7	0.084	0.036	2.331	0.025
	Day 28 –Day 0	0.063	0.028	2.227	0.033
*Bacteroides ovatus*	Day 28 –Day 0	0.080	0.035	2.291	0.029
	Day 28	0.089	0.039	2.27	0.029

Results from linear regression analysis after adjusting for baseline levels of the IgA titers.

*Represents the timepoint of taxa presence or the range of timepoints during which taxa presence changed.

The models used to identify OTU associations with H1 titers were repeated for H3 antibody titers and detected significant associations with several bacterial genera; however, we were unable to positively assign species-level identification for these groups (data without species-level identification not shown).

### 3.5 Multivariate analysis of day 28 immune response

Multivariate models were constructed using penalized regression. The final models for predicting influenza H1 and H3 IgA antibody response at Day 28, based on the bacterial taxa on Day 28, included *B*. *ovatus* presence on Day 28 post-immunization, which was associated with an increase in both H1-specific IgA antibody titers (coefficient = 0.042) and H3-specific IgA antibody titers (coefficient = 0.074). In addition, *S*. *geniculate* presence on Day 28 was associated with a decrease in H3 IgA antibody response on Day 28 (coefficient = -0.031).

## 4. Discussion

Studies have demonstrated the importance of commensal bacteria in immune responsiveness to influenza vaccination and infection in various ways, including the positive impact of probiotic supplements on the immune system [28,29,30,31]. For example, the poor response to influenza vaccination observed in antibiotic-treated TLR5^-/-^ mice can be ameliorated by oral reconstitution of flagellated *E*. *coli* [9]. Probiotic supplementation with *E*. *coli* tends to provoke increased production of the pro-inflammatory cytokines IL-1β, IL-6, TNF-α, GM-CSF, and MIP-1α, along with the anti-inflammatory cytokine IL-10, in a strain-specific manner [32]. Cytokine production by antigen presenting cells, including dendritic cells, monocytes, and B cells, has also been demonstrated to increase with probiotic supplementation [32]. Furthermore, impaired innate and adaptive antiviral immunity, including decreased expression of genes linked to interferon activation and antiviral immunity, was detected in mice that received antibiotic treatment against commensal bacteria prior to exposure to influenza virus [6].

Two commensal bacterial species found to be significantly correlated with influenza-specific IgA titers in our studies include *L*. *helveticus* and *B*. *ovatus*. As discussed below, the bacterial species of the *Lactobacillus* genus are some of the most commonly studied commensals in regard to immunomodulation of the body’s response to influenza infection and vaccination. One clinical trial demonstrated that treatment with *Lactobacillus rhamnosus* GG for 28 days before LAIV administration enhanced the development of H3N2, but not H1N1 or B-strain, seroprotection [7]. Another trial demonstrated that pretreatment with probiotics (*Bifidobacterium animalis* and *Lactobacillus paracasei*) for six weeks before intramuscular influenza vaccination (containing A/H1N1, A/H3N1, and B strains) led to significantly higher vaccine-specific IgG and salivary IgA antibody responses [33]. *Lactobacillus casei* has been studied in mice as an adjuvant for a mucosally administered influenza vaccine. The use of this adjuvant not only resulted in heightened systemic and mucosal protective immune responses, but also broadened protection against divergent influenza subtypes [34,35]. Oral administration of the same bacterial strain in mice mitigated influenza virus infection by significantly lowering viral titers detected in nasal washings and significantly increasing survival rate after infection in two different studies [36,37]. One of these studies demonstrated increased NK cell activation in the respiratory tract, which is hypothesized to be a result of the increased IL-12 production in the mice fed with *L*. *casei* [37], while the other study demonstrated increased IFN-γ and TNF-α production in the mice fed with *L*. *casei* [36]. Another study evaluated nasal administration of a different *Lactobacillus* species–*L*. *rhamnosus*–and demonstrated a resultant increase in resistance to RSV challenge through the activation of TLR3/RIG-I in the respiratory tract [38]. To our knowledge, no studies have specifically evaluated the effects of *L*. *helveticus*, the strain found to be significantly correlated with H1 influenza IgA antibody titers in our studies, on the immune response to influenza vaccination. However, as mentioned above, studies of the *Lactobacillus* genus as a whole with respect to immunomodulation, have shown stimulation of the production of pro-inflammatory cytokines including IL-12 and IFN-γ [32]. Furthermore, *Lactobacillus* strains tend to produce a high ratio of TNFα:IL-10 in PBMCs from healthy subjects 58–65 years old [32], which is indicative of a strong pro-inflammatory response.

Interestingly, *L*. *helveticus* only showed a significant correlation with influenza H1 IgA antibody response and only by univariate analysis, while *B*. *ovatus* correlated significantly with H1 IgA antibody response by both univariate and multivariate analyses and with H3 IgA antibody response by multivariate analysis. Other studies examining the effects of administration of various *Lactobacillus* strains on influenza H1- and H3-specific IgA antibody titers post-influenza vaccination show differences in levels of correlation between the probiotic strain of interest and the two influenza antibody titers [39,40]. This may be due to complementary effects of the immune response mechanisms between the probiotic strains and proteins of interest; however, more studies are needed to validate this speculation. We are unaware of studies evaluating the effects of *B*. *ovatus* on influenza H1 or H3-specific IgA antibody titer after influenza vaccination. However, the *Bacteroides* genus as a whole has been demonstrated by 16s rRNA sequencing to have a modest, but statistically significant, increase in relative abundance 1–2 weeks after LAIV administration in healthy subjects 18–65 years old [41].

Other species correlated (either positively or negatively, as indicated) with influenza titers in our studies include the commensal strains *P*. *melaninogenica* (positive), *V*. *dispar* (negative), and *S*. *infantis* (positive). There are several studies demonstrating that two of these species–*P*. *melaninogenica* and *V*. *dispar*–are related to respiratory health. One study used 16s rRNA sequencing to compare the oropharyngeal microbiota of pneumonia patients with that of healthy patients, using a young and an elderly cohort for each group, and found that *P*. *melaninogenica* and *V*. *dispar* were more prevalent in the healthy subjects from both cohorts. [42] Our results demonstrated a negative correlation between influenza-specific H1 IgA titers and *V*. *dispar* presence at Day 0. While these results may seem contradictory to those in the aforementioned study, it may also be an effect of the differences in study design; our study evaluated the effect of the presence of bacterial species on influenza-specific immune response in healthy patients only, while the former compared the microbiota of healthy versus patients with pneumonia. In a study of bronchoalveolar lavage (BAL) samples from ICU patients with pneumonia compared to ICU patients without pneumonia, the amplification of 16S rDNA genes showed that *P*. *melaninogenica* was significantly correlated with the lack of pneumonia [43]. Another study found *P*. *melaninogenica* to be more prevalent in the oropharynx of healthy control subjects compared to those with asthma or COPD [44]. The results of these studies, together with the fact that these species are associated with influenza H1 IgA antibody titers in our study, indicate that *P*. *melaninogenica* and *V*. *dispar* are important for overall respiratory health. Studies evaluating the immunomodulatory effects of *S*. *infantis* are quite limited and do not offer sufficient information to allow for further interpretation of our results [45]; however, the *Streptococcus* genus has been demonstrated to be in increased abundance in healthy individuals compared with those with respiratory infections [46]. Our results suggest that multiple bacterial strains may exert immunomodulatory effects in the nasal airway. Follow-up studies will be necessary to fully understand interactions between the microbiome and the host during the development of immune responses during infection or vaccination.

The main strength of this study is that, to our knowledge, it is the first study to investigate LAIV-induced alterations to the human nasal microbiome and the subsequent immune-related effects of these alterations. An additional strength is the use of next-generation 16S rRNA sequencing in order to begin delineating the relationship between vaccination with LAIV and the human nasal microbiome. Our results show that alterations of the nasal microbiome do, indeed, occur after LAIV vaccination, and that some of these alterations are correlated with influenza-specific IgA response. Specifically, a significant increase in alpha diversity occurs between Day 7 vs Day 0 post-vaccination and between Day 28 vs Day 0 post-vaccination when measured by observed number of OTUs, ACE estimator, and Chao’s estimator; however, when utilizing Shannon diversity index to measure richness and evenness we do not observe significant differences in diversity. As ACE and Chao estimate richness (the number of species present), and while Shannon measures both richness and evenness (the degree to which individuals are split among species),[47] our results suggest that vaccination affects the number of species present rather than the relative abundance of those species. The primary weakness of this study was the recruitment of a small sample size, which limits our ability to detect smaller effects; for this reason, we chose to present actual p-values, rather than defining a significance cutoff or adjusting for multiple testing.

In conclusion, our study demonstrates significant increases in the richness of the nasal microbiota after intra-nasal influenza vaccination. Other studies have demonstrated increased bacterial growth and persistence of bacterial carriage in the upper respiratory tract after LAIV, [48] increased susceptibility to bacterial infections, [48,49] and changes in relative abundance of certain bacterial species [49] in mice after influenza virus infection. While the specific mechanisms leading to increased species richness following intra-nasal administration of LAIV–along with the mechanisms impacting immune response–require further investigation, it has been suggested that activation of the type-III interferon response after influenza infection has an effect on increased susceptibility to lower respiratory bacterial infections and enhanced proliferation of the microbiome in the upper respiratory tract [49]. It should also be noted that decreased bacterial richness tends to be associated with increased adiposity, insulin resistance, dyslipidaemia, and inflammatory phenotypes [50]–all characteristics that are known to negatively impact immune response. This data aligns with the data of many of the aforementioned studies, which evaluate the presence of the particular bacterial species our studies show to be positively correlated with antibody titer after LAIV receipt and demonstrate increased abundance of these species in healthy patients versus those with respiratory infections after influenza vaccination or infection.[7,33,34,35,36,37,42,43,44,46]

We also identified significant associations between influenza-specific H1 and/or H3 IgA antibody titers and several bacterial species. Further studies using systems biology approaches to understand the mechanisms that allow for increased species richness after vaccination, as well as the colonization of the particular bacterial species associated with increased influenza-specific titers post-vaccination, could significantly advance the field of vaccinomics [51,52,53,54], leading to methods of intensifying the immune response to LAIV, the development of novel vaccine candidates, enable more personalized vaccine approaches, and potentially increase the overall vaccine efficacy for this economically and immunologically burdensome disease.
